# Channel Network Control on Seasonal Lake Area Dynamics in Arctic Deltas

**DOI:** 10.1029/2019GL086710

**Published:** 2020-03-31

**Authors:** Lawrence Vulis, Alejandro Tejedor, Jon Schwenk, Anastasia Piliouras, Joel Rowland, Efi Foufoula‐Georgiou

**Affiliations:** ^1^ Department of Civil and Environmental Engineering University of California Irvine Irvine CA USA; ^2^ Department of Science and Engineering Sorbonne University Abu Dhabi Abu Dhabi United Arab Emirates; ^3^ Earth and Environmental Sciences Division, Los Alamos National Laboratory Los Alamos NM USA; ^4^ Department of Earth System Science University of California Irvine Irvine CA USA

**Keywords:** arctic deltas, permafrost, remote sensing, lakes, arctic hydrology

## Abstract

The abundant lakes dotting arctic deltas are hotspots of methane emissions and biogeochemical activity, but seasonal variability in lake extents introduces uncertainty in estimates of lacustrine carbon emissions, typically performed at annual or longer time scales. To characterize variability in lake extents, we analyzed summertime lake area loss (i.e., shrinkage) on two deltas over the past 20 years, using Landsat‐derived water masks. We find that monthly shrinkage rates have a pronounced structured variability around the channel network with the shrinkage rate systematically decreasing farther away from the channels. This pattern of shrinkage is predominantly attributed to a deeper active layer enhancing near‐surface connectivity and storage and greater vegetation density closer to the channels leading to increased evapotranspiration rates. This shrinkage signal, easily extracted from remote sensing observations, may offer the means to constrain estimates of lacustrine methane emissions and to develop process‐based estimates of depth to permafrost on arctic deltas.

## Key Points

- Summertime lake shrinkage in arctic deltas is highly structured around the channel network
- Higher shrinkage rates closer to channels are attributed to enhanced near‐surface flow and storage as well as greater vegetation density
- Lake methane emission estimates on arctic deltas should account for spatially and seasonally variable lake extents to reduce uncertainty

## 1 Introduction

Lakes play a key role in the hydrologic and biogeochemical cycles of arctic deltas, serving as hotspots of methane and carbon dioxide emissions (Squires et al., 2009; Tank et al., 2009). Thus, understanding lake response to permafrost thaw and constraining lacustrine emission estimates is critical for forecasting trajectories of the polar north (Elder et al., 2018; Wik et al., 2016) as arctic deltas alone are estimated to contain 90 ± 37 PgC (Schuur et al., 2015), compared with 860 PgC in the atmosphere (Le Quéré et al., 2018). Annual methane emissions from lakes have been estimated using a nonlinear dependence on lake area, typically computed using mean annual lake area (Bastviken et al., 2004). However, remote sensing studies of arctic lake area dynamics across various geomorphic settings and on seasonal to annual time scales have found seasonally variable lake area extent depending on proximity to river source, lake morphometry, and underlying permafrost content (Chen et al., 2012, 2013; Jepsen et al., 2013; Rey et al., 2019; Rover et al., 2012; Smith et al., 2005). For example, Cooley et al. (2019) found that in 2017 on the Mackenzie Delta, lake areas had decreased from their June maximum by 8–12% in August. This temporal variability contributes to uncertainty in the arctic carbon budget, especially given the nonlinear dependence of methane emissions on lake area. Lake and wetland coverage also modulate surface albedo, therefore understanding variable water extent is key for modeling the land surface energy budget of the Arctic (Vonk et al., 2015). In addition, long‐term trends of lake area extent over the past several decades are highly spatially heterogeneous, and seasonal variability in lake sizes is of the same order of magnitude as observed decadal trends (Chen et al., 2013), indicating the need to understand seasonal heterogeneity for trend quantification and uncertainty reduction of energy and carbon budgets.

Physical drivers of spatial variability in lake area dynamics include depth to permafrost, as the negligible hydraulic conductivity of frozen soil limits groundwater flow, inhibiting hydrologic connectivity between lakes and their surroundings (Walvoord & Kurylyk, 2016). For example, remote sensing studies on the Yukon Flats floodplains found that thicker active layers and associated near‐surface (i.e., shallow) hydrologic connectivity were linked with higher rates of lake area loss and interannual variability in lake area for some lakes closer to the channels, compared with lakes surrounded by shallow permafrost farther from the channels (Rey et al., 2019). Furthermore, field observations on the Colville Delta, Yukon‐Kuskokwim Delta, and other arctic floodplains have found that the river network imposes a pattern on the permafrost and vegetation distribution on the delta through repeated flooding and inundation of the areas closer to channels, leading to a thicker active layer (Viereck, 1973; Zheng et al., 2019) and denser vascular vegetation (Jorgenson, 2000; Jorgenson et al., 1997), which may drive patterns in seasonal lake area change. In addition, higher sedimentation rates in lakes closer to the channels may also contribute to systematically shallower lakes (Jorgenson et al., 1997), which would then exhibit higher rates of summertime shrinkage under equal evaporation rates.

We propose that rigorously quantifying from remotely sensed data the seasonal dynamics of lake area extents as a function of their positions relative to the channel network will add valuable insight into the hydro‐geomorphologic functioning of these systems, which is hard to directly measure in the field. It will also provide a means for improved estimates of carbon and energy fluxes, which are nonlinearly dependent on lake area extents. In this paper we present a detailed analysis of summertime lake shrinkage in two arctic deltas (Yukon and Colville) as a function of the distance to the nearest channel and document a highly structured variability which is specific to each delta, providing a signature of the system's hydro‐geomorphologic structure and seasonal dynamics. Through consideration of possible explanatory variables (surface connectivity, temperature, and vegetation spatial structure) we propose predominant physical mechanisms for the observed patterns and suggest the attractive possibility of using remote sensing observations of lake area seasonal change to augment mechanistic understanding of arctic hydro‐geomorphology.

## 2 Deltas, Data, and Channel Network Extraction

**Figure 1:**
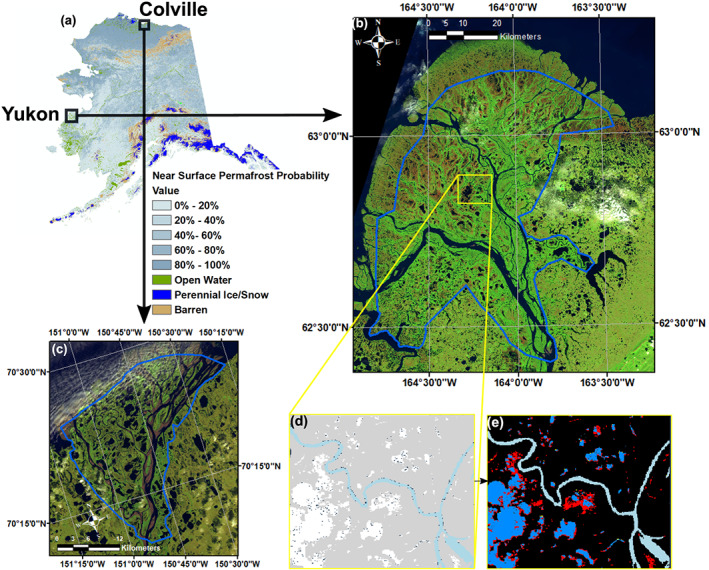
Study areas and illustration of seasonal lake area shrinkage. (a) A map of the near‐surface permafrost probability from Pastick et al. (2015) and the locations of the Colville and Yukon deltas. (b) A Landsat 8 scene (falsely colored in R‐Surface Water Infrared, G‐Near Infrared, B‐Green) taken on 6 July 2014 over the Yukon Delta, with the study zone outlined in blue. (c) The same over the Colville Delta on 12 August 2014. (d) The classified June 2008 water mask from the Global Surface Water (GSW) data set, with land in gray, channels in light blue, lakes in white, and no data in dark gray. (e) The lake area shrinkage from June to July 2008 is depicted with water that drained or evaporated marked in red, water that remained water in dark blue, and land shown in black.

We studied the summertime lake area dynamics of two Alaskan deltas: the Yukon (apex at 62°N, 3,415 km^2^, discontinuous permafrost zone) and the Colville (apex at 70°N, 549 km^2^, continuous permafrost zone) (Figure 1). The two systems have different climates, riverine sediment characteristics, and morphologies. The Yukon is characterized by abundant lakes but a lack of permafrost features such as polygonal tundra, a sediment load primarily of silty and sandy material (Dupre & Thompson, 1979), a mean annual air temperature of −1.2 °C, and a mean summer air temperature of 11.6 °C (NOAA GSOD Station 702005). The Colville is characterized by numerous lakes and permafrost features including ice‐wedges and frost mounds, and has a sediment load of mainly sand and gravel (Walker, 1998), mean annual air temperature of −11.0 °C, and mean summer air temperature of 6.2 °C (NOAA GSOD Station 700637). Estimates of near‐surface permafrost presence indicate a 98% chance of observing permafrost within 1 m of the surface on the Colville (Pastick et al., 2015, Figure 1a), with active layer thickness between 36 and 100 cm, and vegetation composed of willows, sedges, and bryophytes (Jorgenson et al., 1997). This is in contrast with the 17% chance of near‐surface permafrost on the Yukon (Pastick et al., 2015, Figure 1a), indicating active layer thickness in excess of 1 m and/or the complete absence of permafrost, and vegetation composed of willows and sedges (Jorgenson, 2000).

To analyze lake area changes on the deltas, we used the Global Surface Water (GSW) data set which provides monthly 30‐m spatial resolution, Landsat‐derived global water cover masks from March 1985 to December 2018 (Pekel et al., 2016). We present analysis of the monthly lake shrinkage rates from June to July (summer), the months which have the greatest data availability and correspond to the period post‐snowmelt and streamflow recession (see supplementary information for further information, Figure S1). Data availability and quality (e.g., missing data due to clouds, Landsat 7 striping, and snow cover) are variable during the time of record, so we applied a threshold of at least 60% of the delta (excluding the channel network) being resolved (i.e., classified as land or water), which was met for 10 summers on the Yukon and for 16 summers on the Colville (Figure S2). To compare lake shrinkage rates from year to year, we used the Interactive Multisensor Snow and Ice Mapping System 24‐km daily snow cover data set, available from 1997 till present, to compute the date of snow cover disappearance as the date when less than 15% of the study area was classified as snow (Helfrich et al., 2007; National Ice Center, 2008).

Computing lake area change as a function of the distance to the nearest channel required first extracting the Delta Channel Network (DCN) and second computing the distance of every pixel (land or water) to the nearest channel. Automatic channel network extraction for river deltas has recently been advanced with the Python package RivGraph (Schwenk et al., 2020), which utilizes water coverage imagery to extract and skeletonize the DCN. We found that no major channels avulsed or lake breaching took place on either delta during the period of record and therefore used a constant DCN (see the supporting information for more details). To reduce the effect of channelized flow, only water bodies (e.g., inundated depressions, ponds, and lakes, hereafter collectively referred to as lakes) disconnected from the channel network were considered in our analysis. We also utilized higher resolution DigitalGlobe imagery to account for small streams not visible in Landsat (section 4). Once the DCN was extracted and the disconnected lakes were identified, we computed the shortest linear distance of every land or water pixel to the nearest channel, *d*_nc_, a calculation equivalent to the distance transform of a binary image (Haralick & Shapiro, 1991). The probability distribution functions of *d*_nc_ for both deltas are provided in the supporting information (Figure S2).

## 3 Summertime Lake Area Dynamics as a Function of Distance to the Nearest Channel

**Figure 2:**
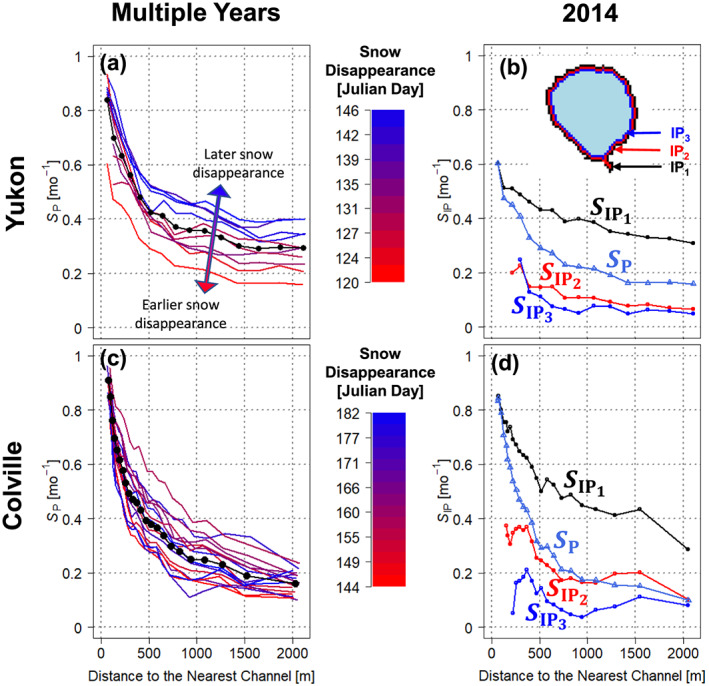
Summertime lake shrinkage as a function of the distance to the nearest channel. (a, c) Shrinkage rate, estimated by the monthly fraction of water area loss *S*_*P*_ as a function of *d*_*nc*_, for 26 summers, each curve marked by the date of snow disappearance on the Yukon (a) and the Colville (c) with the weighted average shrinkage rate curve shown in a black dotted line. (b, d) The results of the lake shoreline shrinkage from an object‐based analysis are shown for 2014 on the Yukon (b) and for 2014 on the Colville (d), with the pixel‐based estimate shown in light blue as comparison. The inset in (b) highlights the first three Internal Perimeters (IPs) of a sample lake in black, red, and blue, with the remaining water shown in light blue.

We tested the control of the DCN on summertime lake area dynamics by quantifying the lake shrinkage rate, S, using a pixel‐based monthly shrinkage estimate, *S*_p_, as a function of *d*_nc_. *S*_p_ is computed as the fraction of water area loss from 1 month to the next, in our case from June to July (see description of data availability and calculations in the supporting information). We found that both deltas, for every year in the period of record (2001–2018), showed a robust pattern in lake shrinkage rates (Figures 2a and 2c) with a systematic decrease of shrinkage rates farther away from the channels, reaching almost a constant rate after a distance characteristic of each delta (approximately 1,500 m for Yukon and 2,000 m for Colville). The interannual variability in the magnitude of *S*_p_ was identified as being associated with differences in the day of snow cover disappearance, with a higher shrinkage rate when snow cover disappeared later in the year, in our case closer to June; see Figures 2a and 2c. As the shrinkage pattern seems robust from year to year and is modulated only in magnitude, we estimated the weighted average normalized water area loss, $\bar{S_{p}}$, as a function of *d*_nc_, shown in black in Figures 2a and 2c, where the weights were proportional to the number of valid (i.e., resolved) pixels each year in each distance bin. The observed summertime shrinkage signal is apparent in over 26 years of data over the two deltas, indicating that the topology and geometry of the channel network leave a signature on the spatial pattern of lake shrinkage.

While *S*_p_ can be computed in summers with missing data (e.g., due to cloud cover), this pixel‐wise approach neglects the spatial context of the water pixels, i.e., lumps all water pixels together irrespective of their arrangement within specific water bodies. We know, however, that the location of a water pixel, e.g., an interior versus exterior pixel of a lake, is subject to different hydro‐geomorphologic processes and thus analyzing shrinkage rates within an object‐based context can provide complementary and physically interpretable information. This object‐based approach, however, can only be performed on imagery with sufficient data quality (i.e., with a negligible fraction of no data pixels). To implement an object‐based approach we used an iterative morphological erosion operation (see Figure 2 inset; Haralick & Shapiro, 1991) to classify water pixels according to their position in the different lakes. Specifically, we classified them as belonging to an internal perimeter (IP), with IP_1_ indicating the shoreline perimeter of a lake, IP_2_ the next internal perimeter, etc. We then estimated a monthly lake shoreline shrinkage rate, *S*_*IP*__*i*_, computed as the fraction of water area loss of water pixels in IP_*i*_, as a function of *d*_nc_. We note that the weighted average of *S*_*IP*__*i*_ for all *i*, with weights proportional to the number of IP_*i*_ pixels over the total number of water pixels, will converge to *S*_p_ when all IPs are considered, i.e., when the morphological erosion operation has “eroded” all lakes to their center‐point. We highlight the results of this object‐based analysis for the first three IPs on the summer of 2014, when both deltas had over 99% of the nonchannel study area resolved, and compare them with the pixel‐based analysis results made over multiple years.

The shrinkage rate of the shoreline perimeter of lake bodies (IP_1_) for year 2014 shows a well‐behaved decreasing pattern as a function of *d*_nc_ (Figures 2b and 2d). On the Yukon, a steady but slower decrease in *S*_IP__2_ and *S*_IP__3_ is observed compared with $S_{{\mathrm{IP}}_{1}}$. On the Colville, $S_{{\mathrm{IP}}_{2}}$ and *S*_IP__3_ decrease with *d*_nc_ albeit they are more variable. Independent of distance, as expected, the most external IPs have a higher rate of shrinkage, but decay to similar distances compared with the pixel‐based shrinkage rate, S_p_. Given that this method is a more direct representation of lake shrinkage compared to the pixel‐based approach and that similar patterns and length scales of shrinkage rates are identified, this result strongly supports the existence of a spatial organization of lake shrinkage rates around the channel network. It also validates the use of a pixel‐based estimate which enables lake shrinkage rates to be quantified more readily even when there is missing data in the water masks.

## 4 Physical Attribution of the Spatial Pattern of Summertime Shrinkage

We explored three physical mechanisms that may contribute to the observed spatial pattern of shrinkage: surface connectivity of lakes closer to the channels via very narrow pathways not detected in the Landsat imagery of 30‐m resolution, systematically shallower lakes closer to the channels versus farther away, and enhanced vegetation coverage and a thicker active layer closer to the DCN.

### 4.1 Drainage Due to Subpixel Surface Connectivity

At the 30‐m spatial resolution of GSW, unresolved structural connectivity, e.g., narrow tie channels (Rowland et al., 2009), may lead to the mischaracterization of lakes as disconnected. The higher shrinkage rates may then be due to these subpixel channels, and therefore the decreasing shrinkage rate a signature of sub 30‐m DCN structure. To test this, we randomly sampled between both deltas a total of 1,069 lakes identified from the GSW imagery and used DigitalGlobe (0.31 to 0.65 m spatial resolution) imagery available via Google Earth to identify subpixel channel connectivity (see supplementary information for dates). Channel‐lake connectivity was manually determined based on the observed presence or absence of small, connected channels over the summer months (June to August) as in Chen et al. (2013). On the Yukon we sampled 809 out of 12,745 lakes in 2014, and found 547 lakes disconnected from the channel network. On the Colville we sampled a total of 260 out of 1,409 lakes in 2014, and found 211 disconnected.

**Figure 3:**
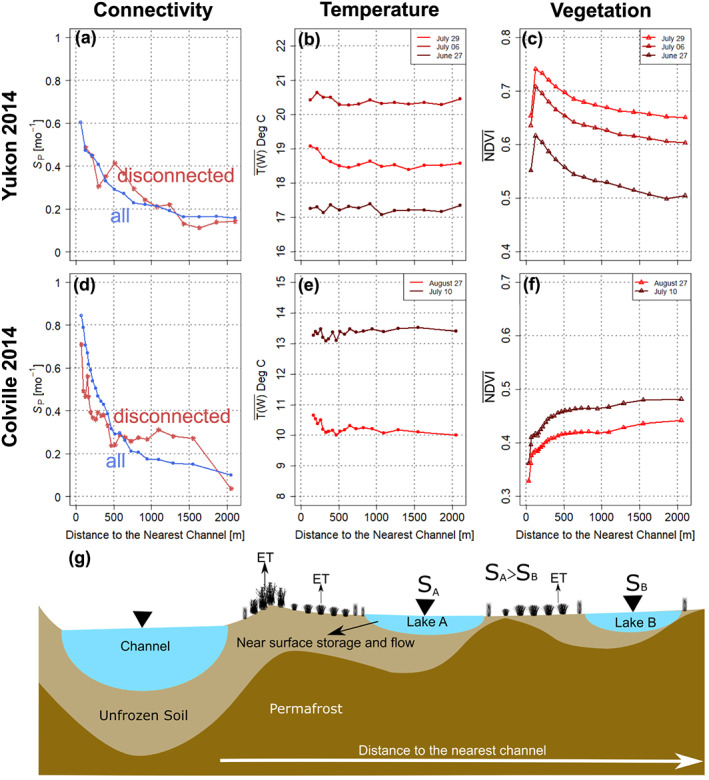
Higher resolution figure attached as PDF.Examining physical mechanisms for increased lake shrinkage closer to the delta channel network. (a, d) Comparison of the 2014 water area shrinkage rates of all lakes disconnected from the channel network as inferred from the Landsat images at 30‐m resolution (same as in Figures 2a and 2b) and a subset of lakes disconnected from the channels as inferred from high resolution (0.6 m) to rule out that subpixel surface connectivity not seen in Landsat cannot explain the observed structured shrinkage patterns. (b, e) Average surface temperature of water pixels in internal perimeter IP_2_ (a proxy for lake depth) is independent of distance from the channel network indicating that lake depth is not the primary cause for the observed higher shrinkage rates closer to the channels. (c, f) Mean NDVI of June land pixels spikes and decreases on the Yukon (c), and steadily increases on the Colville indicating presence of barren sandbars next to the channels (f). (g) Schematic illustrating that the enhanced shrinkage, *S*, closer to the DCN is predominantly caused by increased near‐surface storage and flow, a result of increased heat content near the channel, and modulated by higher evapotranspiration rates due to denser vegetation content on the Yukon.

To evaluate the DCN control on shrinkage rates we used the GSW imagery to compute *S*_*p*_ for the subsampled lakes identified as disconnected at the DigitalGlobe 0.6‐m resolution. We found that these lakes exhibited decreasing *S*_*p*_ as a function of *d*_nc_ up to 1,500 m (Figures 3a and 3d), albeit the patterns are less smooth due to the small sample sizes. These results indicate that the higher shrinkage rates closer to the DCN are not the result of surface connectivity by narrow (<30 m width) channels, but rather the result of near‐surface hydro‐geomorphologic processes.

### 4.2 Systematic Control on Lake Depth as a Function of Distance to the Nearest Channel

Lakes located closer to the DCN in general have higher inundation and sedimentation rates than lakes farther away, as indicated by the classification of Jorgenson (2000), and therefore might be shallower (i.e., lower height to width ratios closer to the DCN). Systematically shallower lakes closer to the DCN could then have higher shrinkage rates, even with equal evaporation rates. Here we wanted to test the hypothesis that systematic lake depth increase with distance is not the primary cause for the observed higher shrinkage rate closer to the channels. However, there is a lack of delta‐wide lake bathymetry measurements to validate this hypothesis. As a proxy to bathymetry, we used water surface temperatures, positing that shallower lakes will likely have warmer surface temperatures than deeper lakes. We used Landsat Thermal Infrared (TIR) band‐derived land surface temperature (LST) (Malakar et al., 2018) to analyze surface water temperatures over individual Landsat scenes. To obtain LST data from TIR reflectance, a thermal emissivity *ε* for each pixel must be specified, which is constant over water. To discard water LST variability due to emissivity heterogeneity, we analyzed only pixels classified as water both in the GSW and LST data set, i.e., pixels with *ε* greater than 0.99 in the spectral range of the Landsat 8 TIR.

Visual inspection of the Landsat scenes showed that the centers of lakes do not show significant temperature variability, likely due to a depth threshold being achieved where the water surface temperature is not primarily controlled by lake depth. To account for differences in lake morphology and for the fact that lakes are generally smaller closer to the channel network (Figure S4), we analyzed the temperature on the outer edges of the lake (e.g., IP_2_). We found that the average outer perimeter water temperature was nearly constant, i.e., independent of *d*_nc_ (Figures 3b and 3e). As we explicitly account for the position of a water pixel relative to the edge of the lake it lies in, this analysis specifically tests whether there are systematically warmer, and thus shallower, lake banks closer to the DCN. The nearly constant temperature observed across the edges of lakes on the deltaic surface does not support the hypothesis of systematically shallower lakes closer to the DCN, implying that lake depth is not the primary control of the observed shrinkage pattern.

### 4.3 Enhanced Near‐Surface Connectivity and Vegetation Density by the DCN

Extensive field studies on the Colville and the Yukon‐Kuskokwim delta have found that in permafrost affected fluvial landscapes, the coevolution of landforms, permafrost, and vegetation imprints distinct spatial patterns on the geomorphology and ecology of the landscape (Jorgenson, 2000; Jorgenson et al., 1997; Shur & Jorgenson, 2007). These studies have documented a gradient in vegetation density and type with distance from the channels, with barren sandbars immediately around the channel network, followed by vascular vegetation including willows and shrubs in the active floodplain around the DCN, compared with greater sedge and bryophyte density in the inactive floodplain farther from the DCN, indicative of deeper active layer thickness near channels (Jorgenson, 1998, 2000; Jorgenson et al., 1997). In the active floodplain, higher rates of sedimentation limit organic matter deposition and permafrost aggradation, while the inactive floodplain has a thicker organic layer, which insulates and protects frozen soil contributing to shallower depth to permafrost (Jorgenson, 1998). On the Colville, visual inspection showed that the channel network is abutted by barren sandbars, while on the Yukon the channel network is generally enveloped by land cover with high near infrared reflectance, colored in green in Figure 1b, indicative of greater vegetation density and photosynthetic activity (Laidler et al., 2008). Given greater sand deposition immediately around the DCN and enhanced vegetation content on the floodplain closer to the DCN (Jorgenson, 1998), greater shrinkage rates may be due to greater subsurface flow pathways (due to deeper active layers) and higher evapotranspiration rates (Figure 3g). Due to a paucity of field observations of subsurface flow and evapotranspiration rates spanning the spatial and temporal domains analyzed, we examined DCN control on vegetation coverage using Landsat‐derived Normalized Difference Vegetation Index (NDVI) as a proxy for vegetation density and therefore evapotranspiration rates.

Individual Landsat scenes from June to August 2014 over both deltas were used to compute the mean NDVI of land, as identified from the June 2014 GSW water mask, as a function of *d*_nc_ (Figures 3c and 3f). On the Colville, an increase in NDVI with *d*_nc_ is observed for both Landsat scenes, which is due to the sparsely vegetated sandbars adjacent to the channels, that undergo frequent ice scouring and reworking (Jorgenson, 1998), thus significantly decreasing the mean NDVI. On the Yukon, a sharp increase in NDVI followed by a decrease until 1,500 m from the channel is observed, which corresponds to some sandbars present on the edge of the DCN, followed by dense vegetation which decreases as a function of d_nc_. These interpretations are consistent with field survey photos provided by N.J. Pastick; see also Pastick et al. (2014). The overall average NDVI in the colder Colville delta is relatively lower than in the warmer Yukon delta and indicates sparser vegetation and lower photosynthetic activity. However, as indicated by transects from field surveys done on the Colville between 1992 and 1996, the deepest thaw depth and coarsest soil is located in these sandbars (Jorgenson et al., 1997), which implies greater water storage capacity and hydraulic conductivity closer to the DCN. The relatively shallower thaw depths on the colder Colville delta have less near‐surface storage and flow capacity than the warmer Yukon delta, which likely contributes to the steeper gradient in shrinkage rates (Figures 2a and 2c).

The presence of deeper thaw zones closer to waterbodies in the Arctic is supported by numerical modeling and observational evidence (Rowland et al., 2011; Woo, 2012). For example, comparison of a borehole located 6 m versus 145 m from the edge of a fjord in Svalbard showed significantly higher maximum and average temperatures throughout the soil profile at the site closer to the fjord (Kristensen et al., 2008). In addition, heat advection from near‐surface flow accelerates heat transport, preventing permafrost formation or thawing existing permafrost (Aas et al., 2019; McKenzie & Voss, 2013; Rowland et al., 2011; Walvoord & Kurylyk, 2016; Wellman et al., 2013). Empirical estimates of near‐surface permafrost indicate that the probability of observing shallow permafrost increases with distance from the channel network (Pastick et al., 2015; Figure S5). These studies provide evidence that waterbodies and water flow modify subsurface flow pathways via modulation of temperature‐controlled soil permeability; therefore, enhanced shrinkage due to near‐surface connectivity may act as a positive feedback by maintaining a deeper active layer thickness and in turn amplifying near‐surface storage and flow. This effect is likely present near all lakes (Figure 3g), not only near the DCN, and contributes to the length scale of DCN control on shrinkage rates.

## 5 Conclusions

Analysis of the summertime surface lake area dynamics of arctic deltas indicates that lake area extent primarily decreases following snowmelt, and that the monthly shrinkage rate strongly depends on the distance from the channel network, with higher shrinkage rates closer to the channels. This signal is detected every summer over a combined 26 years of satellite observations over two deltas of different climate and morphology. This seasonal lake shrinkage signal should be considered in future estimates of lacustrine methane emissions, e.g., those based on nonlinear relationships between methane emission and lake area (Bastviken et al., 2004), to constrain uncertainties in the arctic carbon budget. Higher rates of decreasing water coverage closer to the channels will imply a spatially heterogeneous but structured distribution of methane emissions within a delta which must be accounted for in regional or global assessments and also in projected trends (Cooley et al., 2019).

The higher shrinkage rates seen closer to the channel network are likely primarily caused by enhanced near‐surface storage and flow closer to the channels and by denser vegetation coverage, a signature of the coevolution of landforms, permafrost, and ecology of these arctic landscapes. As the observed pattern is therefore controlled, in part, by physical processes that are difficult to measure in the field (i.e., near‐surface flow and storage), this analysis presents the potential for partial inference of such processes from targeted analysis of readily available Landsat imagery. The spatial and temporal variability of surface and subsurface hydrologic connectivity of lakes in arctic deltas and future trends under warmer temperatures is of the utmost importance for assessing and constraining estimates of carbon emissions and for providing quantitative metrics of change. For example, permafrost thaw and associated increasing hydrologic connectivity (Walvoord & Kurylyk, 2016) may shift colder systems such as the Colville to experience increased near‐surface flow, altering residence and transport times of water and nutrients on the delta. Future work will expand the analysis to a larger set of arctic deltas (Piliouras & Rowland, 2020), and use connectivity theory (e.g., Tejedor et al., 2018, 2015a, 2015b) to quantitatively study the topology of the complex channel‐lake networks of arctic deltas and their expression on the patterns of seasonal lake shrinkage rates.

## Supporting information

Supporting Information S1
