# Supplementary material for: Channel Network Control on Seasonal Lake Area Dynamics in Arctic Deltas
Source: Geophys Res Lett. 2020 Mar 31;47(7):e2019GL086710. doi: 10.1029/2019GL086710 (PMC7380309; doi:10.1029/2019GL086710)
Supplement: Supplementary file 1 — Supporting Information S1 [file GRL-47-e2019GL086710-s001.pdf]

Geophysical Research Letters

Supporting Information for

Channel network control on seasonal lake area dynamics in arctic deltas

Lawrence Vulis[1], Alejandro Tejedor[1,2], Jon Schwenk[3], Anastasia Piliouras[3], Joel Rowland[3], and Efi Foufoula-Georgiou[1,4]

[1]Department of Civil and Environmental Engineering, University of California, Irvine, Irvine, CA, USA 92697
[2]Department of Science and Engineering. Sorbonne University Abu Dhabi, United Arab Emirates
[3]Earth and Environmental Sciencess Division, Los Alamos National Laboratory, Los Alamos, NM, USA 87544
[4] Department of Earth System Science, University of California, Irvine, Irvine, CA, USA 92697

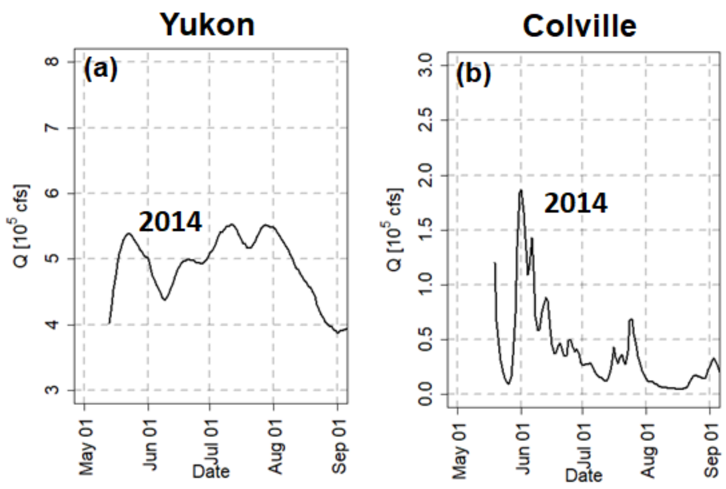

Figure S1. Hydrographs on the Yukon and Colville rivers: (a) Streamflow at Pilot Station (USGS 15565447). The streamgage is downstream of any major confluences and is 75 km away from the apex of the Yukon Delta. (b) Streamflow at Umiat (USGS 15875000). The streamgage is upstream of confluences with the Chandler and Anaktuvuk rivers, and is 100 km away from the apex of the Colville Delta. The bankfull flow, defined as the two-year flow, was estimated on the Yukon from 35 years of data to be 655,000 ft^3/s and on the Colville from 16 years of data to be 176,000 ft^3/s.

Water Mask Description and Channel Network Extraction

Global Surface Water (GSW) masks are images whose pixels may take one of three values: water, land, and no data. No data pixels can arise from lack of imagery, snow and ice cover, cloud cover, and Landsat 7 striping (Pekel et al., 2016). From September to May, no water or land pixels were identified over the Colville (i.e. 100% no data) due to cloud, snow, and ice cover for any year over the period of record. In the Yukon, September to April are unresolved (no data) for all years over the period of record, while some May masks are partially resolved from 2000 to 2018. However, manual inspection of the Landsat scenes used to derive the masks indicate snow and ice cover being resolved as water in parts of the delta, indicating misclassification. Inspection of the summer June and July masks in both deltas indicated that the remaining snow and ice cover were classified as no data or land. We analyzed summers where at least 60% of the delta, excluding the channel network, was resolved as water or land, which included 2002, 2004, 2005, 2007-2009, 2013, 2014, 2016, and 2017 on the Yukon and 2001, 2002, and 2005-2018 on the Colville (see Fig. S2 b, d). Manual inspection of the 2018 June and July masks on the Yukon showed misclassification of large portions of the scene, and this year was not considered in the analysis.

To extract the channel network, we used the Python package RivGraph (Schwenk et al., 2019). RivGraph takes as inputs: (1) a binary mask of the channel network, (2) locations of inlets, and (3) a shoreline, and fully resolves the channel network topology as a set of georeferenced links and nodes. The inlet node was marked at the first major bifurcation of the deltas, and we defined the shorelines excluding the tidal zone, demarcated by lack of vegetation, as seen in Figures 1b and 1c (Dupre & Thompson, 1979; Jorgenson et al., 1997). We used a single water mask, extracted from a composite water mask, where a pixel is defined as water if it shows an 80% June water occurrence over the recorded period. Comparison of the composite DCN skeleton with channel skeletons extracted from individual years did not show significant difference on either delta. To account for the interannual variability in channel extent, for every year analyzed we extracted all waterbodies over the delta using connected component analysis (Haralick & Shapiro, 1991) for each of the two monthly water masks in the summer (June and July) and excluded from our analysis, for the year, any pixels corresponding to connected components (i.e. waterbodies) that overlapped with the DCN. Additionally, for each season, any pixel that was classified as no data in any given month was treated as no data for the entire summer, i.e. a pixel was valid only if it was classified as water or land for June and July in a given year. The remaining objects that were

at least one pixel in size that were disconnected from the channel network were considered as individual waterbodies (i.e. lakes) for each season. Without waterbody bathymetry information, we were unable to systematically remove shallow wetlands during lake extraction, however the water surface temperature analysis shows that it is not more likely for waterbodies closer to the DCN to be shallow wetlands versus waterbodies farther away from channels.

To account for small channels below the Landsat resolution, we used all DigitalGlobe images available via Google Earth for the two deltas to manually identify the presence or absence of surface connections between lakes and the channel network. On the Yukon, the scenes available corresponded to July 16, 2003, June 1, 2005, August 19, 2006, July 9, 2007, September 10, 2008, June 30, 2009, June 5, 2010, July 30, 2010, September 26, 2010, May 25, 2011, August 17, 2011, June 13, 2012, August 9, 2012, September 11, 2012, and October 9, 2012. On the Colville, the scenes available corresponded to July 5, 2005, June 17, 2006, June 29, 2007, August 5, 2011, July 3, 2012, August 11, 2012, August 6, 2013, August 26, 2014, and July 13, 2016. The temporal mismatch between the June and July GSW masks used for extraction of waterbodies and the dates of these high resolution scenes may lead to some misclassification of connected lakes as disconnected, and introduce some uncertainty of our results.

Pixel-based shrinkage calculations:

We estimated the monthly shrinkage rate S, as the pixel-based monthly water area loss fraction S_p using the following methodology. For all land and water pixels we computed the distance to the nearest channel, i.e. d_{nc}, and formed the probability density function (PDF) of d_{nc}, $f(d_{nc})$. We then partitioned the spatial extent of the delta in terms of d_{nc} into K regions where the limits of each region were selected according to equally spaced quantiles of d_{nc}, to ensure that the shrinkage rate was computed from samples of equal size and maintained similar regions from year to year. For every d_{nc} region k, we calculated the fraction of water area lost (i.e. water area that became land area) from time t to time $t+\tau$:

$$S_{p,k} = \frac{A_{w_{k,t} \rightarrow l_{k,t+\tau}}}{A_{w_{k,t}} * \tau} \quad (1)$$

where $A_{w_{k,t}}$ is the water area in region k at time t and $A_{w_{k,t} \rightarrow l_{k,t+\tau}}$ is the water area that became land area in region k at time $t+\tau$; here t corresponds to June and τ is one month. We consider $S_{p,k}$ as an estimate of the shrinkage rate S from June to July for lakes within region k, i.e. lakes located

at distances from the DCN between the q_k and q_{k+1} quantiles of the d_{nc}. As discussed in section 2.3, no major avulsions were observed for the studied deltas during the observational record. This allowed us for each delta to define a constant DCN and the same K regions through time. As the shrinkage pattern appeared robust from year to year and is modulated only in magnitude (Fig. 2a,c), we computed a weighted mean water area loss fraction, $\overline{S_\mathrm{p}}$ for both deltas from June to July over the period of record (Equation 2; Fig. 2a,c dotted black line), where weights $\lambda_{k,y}$ were calculated using Equation 3, where $n_{y,k}$ is the number of valid pixels (water or land) in region k for years y from 1 to Y:

$$\overline{S_\mathrm{p,k}} = \sum_{y=1}^{Y} S_{p,k}\lambda_{y,k} \quad (2)$$

$$\lambda_{k,y} = \frac{n_{y,k}}{\sum_{y=1}^{Y} n_{y,k}} \quad (3)$$

This method treats every water pixel independently of other water pixels, i.e. does not take into account the shape of the lake that the pixel is part of, and therefore has the advantage of not requiring lakes to be completely resolved during the season. Shrinkage rates may thus be estimated even in years with moderate data quality.

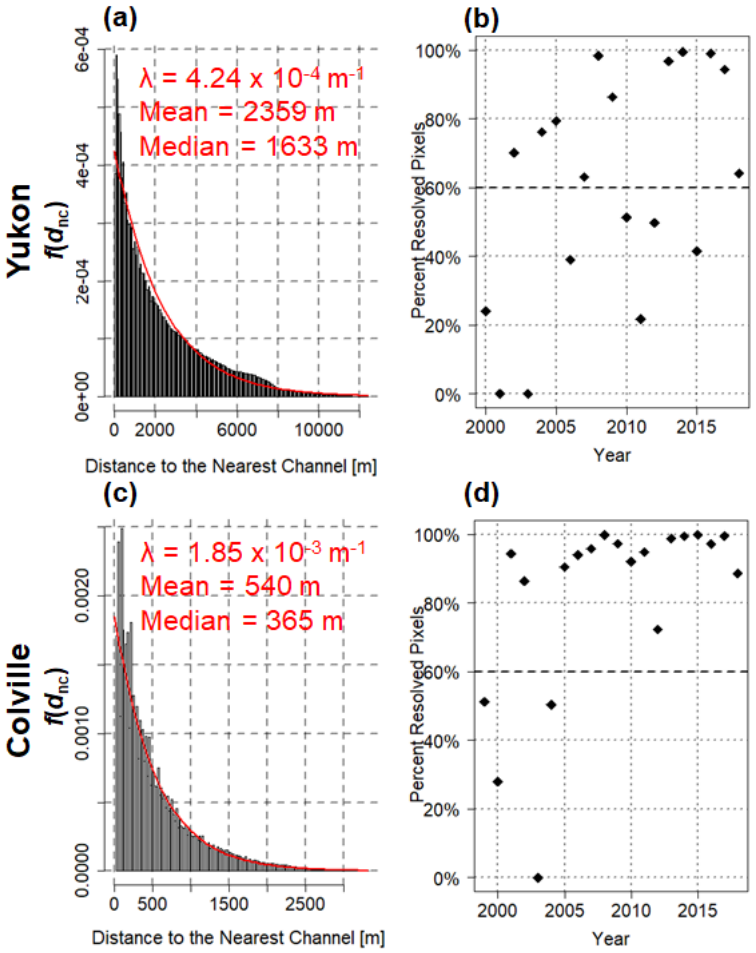

Figure S2. Distance to nearest channel distribution and percent of resolved pixels each year: (a, c) The probability distribution function of nearest distance to the channel network, $f(d_{nc})$ for the Yukon (a) and Colville (c) deltas, with a fitted exponential distribution shown in red. (b, d) The fraction of the delta top resolved in the Global Surface Water dataset for both June and July in each year shown in Figures 2a and c, for the Yukon (b) and Colville (d) deltas, with shrinkage rates only calculated for years with at least 60% of the delta resolved.

Lake Internal Perimeter Definition:

To identify lake shorelines, we used iterative morphological erosion with a diamond-shaped structuring element, which removes a one-pixel thick shoreline, i.e. the ith Internal Perimeter (IP_i), from every object. The eroded water mask is then used as input for the next iteration. The obtained IPs represent the subsequent shorelines of every lake on the delta top. To calculate monthly lake shoreline shrinkage rate at IP_i, S_{IP_i}, equation (1) was used, but only water pixels in the IP_i were used in the calculation. For example, in Figure S3c the fraction of black pixels that shrank would give the monthly lake shoreline shrinkage rate.

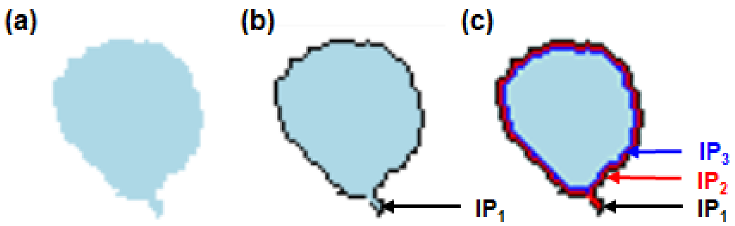

Figure S3. Lake Internal Perimeter Definition: (a) Schematic of Internal Perimeter (IP) extraction using morphological erosion, where classified water pixels are in light blue. Each set of pixels removed by an erosion operation represents a subsequent lake shoreline, or IP. After a single erosion we obtain IP_1 (b). After three erosions we obtain IP_3 (c).

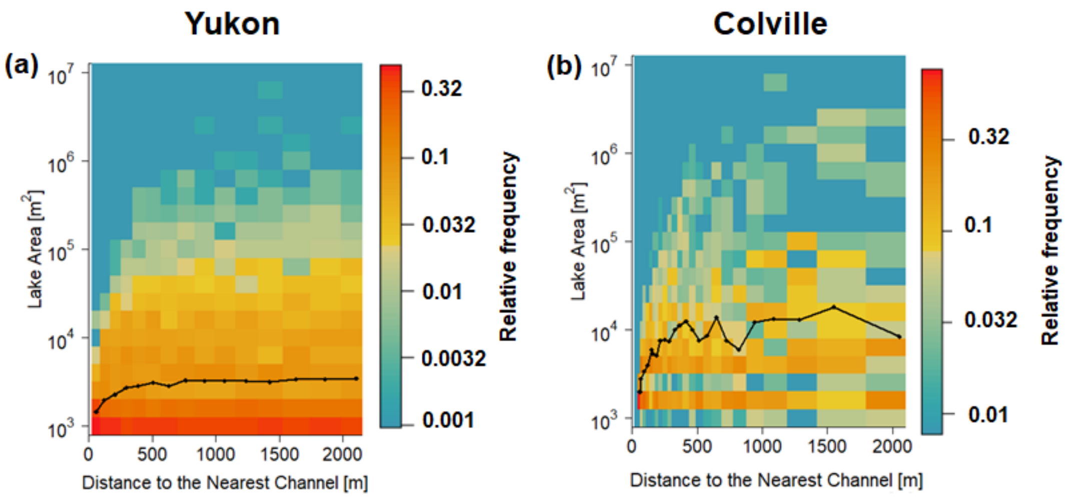

Figure S4. Lake Area Distributions within each distance to the nearest channel region: (a, b) Conditional histograms of object-based lake area within each bin of distance to the nearest channel for the Yukon (a) and the Colville (b) from the Global Surface Water June 2014 water mask, with red-orange colors indicating higher relative frequency. The mean lake area at each distance is indicated by the black line.

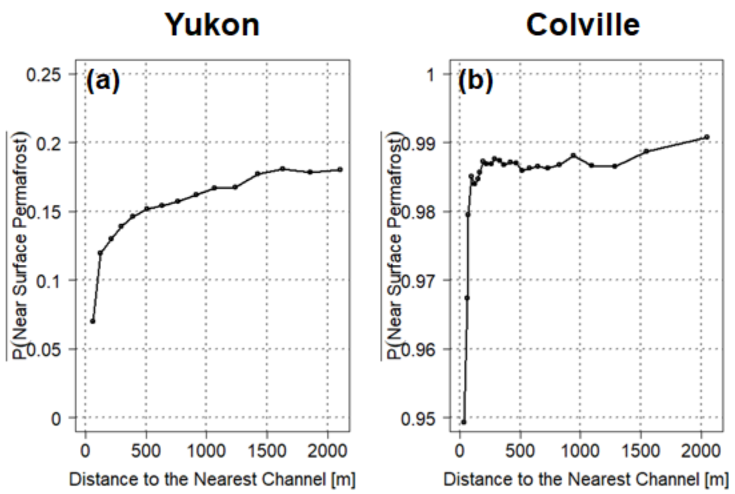

Figure S5. Delta channel network control on near surface permafrost: (a, b) Bin-average probability of observing near surface permafrost, that is, depth to permafrost less than 1 meter extracted from the empirical model of Pastick et al., (2015), versus distance to the nearest channel for the Yukon (a) and the Colville (b). The Yukon shows steadily increasing probability, indicating active layer thickness decreases to less than 1 meter farther from the DCN, while the Colville shows a nearly constant probability, which is supported by observations of thaw depths being on average 30 to 75 cm on the delta (Jorgenson 1998). It is expected that a process-based model of permafrost coverage on an arctic delta should produce similar curves.
